# Lymph node metastasis in lung squamous cell carcinoma and identification of metastasis‐related genes based on the Cancer Genome Atlas

**DOI:** 10.1002/cam4.2525

**Published:** 2019-09-03

**Authors:** Ming Dong, Hao Gong, Tong Li, Xin Li, Jinghao Liu, Hongbing Zhang, Minghui Liu, Gang Chen, Hongyu Liu, Jun Chen

**Affiliations:** ^1^ Department of Lung Cancer Surgery Tianjin Key Laboratory of Lung Cancer Metastasis and Tumor Microenvironment Tianjin Lung Cancer Institute Tianjin Medical University General Hospital Tianjin China

**Keywords:** lung cancer, lymphatic metastasis, squamous cell carcinoma, TCGA database

## Abstract

Squamous cell carcinoma (SCC) is a unique clinical and histological category that accounts for about 30% of total lung cancer. To identify risk factors for lymph node metastasis and analyze the molecular features of these metastases in lung SCC, a retrospective study was performed for 170 lung SCC patients who underwent surgical treatment. The overall survival of these patients with or without lymph node metastasis (LM/NLM) was analyzed using the Kaplan‐Meier method. We also used the TCGA database to compare the differentially expressed genes (DEGs) in patients with stage T1‐2 and T3‐4 lung SCC. Data from both our retrospective study and the TCGA database demonstrated a correlation between age and stage T1‐T2 LM (*P* = .002). There were significant differences between the LM and NLM groups in both mean survival time and median survival time for different T‐stages (*P* = .031). There were 176 upregulated and 177 downregulated DEGs between the LM and NLM groups in the stage T1‐2 group and 93 upregulated and 34 downregulated DEGs in the stage T3‐T4 group. These differentially expressed genes were predicted to participate in five cellular components, five molecular functions, and five biological processes. There were 20 genes, including *GCG, CASR, NPY, CGA, TAC1, ALB, APOA1, CRH, CHRH, TRH,* and *GHSR*, located at the core of the protein‐protein interaction network in the stage T1‐2 group and 11 genes, including *F2, CASR, GRM1, GNRHR, GRPR, NTSR1, PROKR2, UTS2D, PTH, ALB*, and *FGA*, in the stage T3‐4 group. Overall, LM plays a key role in the treatment response and prognosis of SCC patients. Several risk factors, including age and stage, were identified for LM. There was a previously undiscovered enrichment of significant novel genes in lung SCC between the LM and NLM groups, which may have the potential for predicting prognosis and targeting.

## Graphical abstract

Lymph node metastasis plays a key role in the treatment response and prognosis of SCC patients. Several risk factors including age and stage were identified for lymph node metastasis. There is an unprecedented enrichment of significant novel genes in lung SCC between LM and NLM groups, which may have potential for predicting prognosis and targeting.

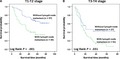

## 1 INTRODUCTION

Lung cancer remains the deadliest cancer worldwide^1^ with a particularly dire prognosis in China.^2^ Squamous cell carcinoma (SCC) of the lung is a unique clinical and histological category of non‐small cell lung cancer (NSCLC) that accounts for about 30% of all lung cancers.^3^ Many of these tumors are in stages IIIA, IIIB, or IV at the time of diagnosis.^4^ Studies have shown that the efficacy of chemotherapy and concurrent chemoradiation for locally advanced NSCLC is unsatisfactory.^5^, ^6^ Although substantial advances have been made in the treatment of non‐squamous NSCLC, effective therapies are still needed for squamous NSCLC. Surgical intervention is the current principal treatment for SCC of the lung, even for locally advanced disease.^7^, ^8^ However, surgical intervention for locally advanced squamous cell lung cancer is still highly controversial because many individual factors affect the surgical outcome. The most important factor is the presence of lymphatic and distant metastasis. In particular, surgical intervention provides a favorable prognosis only for local invasion of the tumor without lymphatic and distant metastasis.

Despite recent advances in the early detection and treatment of lung cancer, the prognosis remains poor partly because of the high rate of recurrence and metastasis after surgical resection. At present, special attention is being paid to lymphadenectomy or lymph node sampling in NSCLC according to China's standards for the diagnosis and treatment of primary lung cancer,^9^ the US national comprehensive cancer network guidelines,^10^ and the European society of thoracic surgeons guidelines.^11^ However, the current situation is not satisfactory because the status of lymph node metastasis is not clear and lymph node biopsies are either not performed or performed carelessly.^12^ For NSCLC that are at a clinically early stage (proven only after surgery), the question remains: “Does complete lymphadenectomy bring unnecessary risks to the patient?” It has been reported that systemic lymph node dissection prolongs the hospital stay and increases postoperative morbidity. Therefore, more and more studies have focused on identifying the risk factors for lymph node metastasis (LM) in clinical early‐stage NSCLC. Identifying these risk factors will help with treatment selection (eg, surgical versus non‐surgical approach; systemic lymph node dissection).

## 2 MATERIALS AND METHODS

### 2.1 Patients

This study was approved by the Ethical Review Committee of Tianjin Medical University General Hospital. All biological samples were obtained with the patients’ written informed consent. All procedures and experimental protocols were approved by the Laboratory Animal Ethics Committee of Tianjin Medical University, and all methods were performed following the relevant guidelines. We retrospectively examined data from patients with squamous cell carcinoma of the lung (LUSCC), who received surgical treatment at the Department of Lung Cancer Surgery, Tianjin Medical University General Hospital between January 2008 and December 2011. A total of 170 patients were enrolled in the study, including 92 patients with stage T1‐T2 and 78 patients with stage T3‐T4 LUSCC. Patients were grouped according to the presence or absence of lymph node metastasis. In the stage T1‐T2 group, there were 57 patients without metastasis and 35 patients with metastasis. In the stage T3‐T4 group, 27 patients had no lymph node metastasis and 51 patients with metastases. Patients treated with surgery for histologically confirmed SCC were considered eligible if they had tissue available for analysis and had clinical follow up data available. The demographic data, complete medical history, pathology results, and follow up data were recorded and verified in real‐time. Survival data were ascertained through medical record review. The TNM stage was classified according to the American Joint Committee on Cancer (AJCC) 2017 8th edition of the tumor node metastasis (TNM) classification using standard radiological guidelines. The staging was carried out using computerized tomography scans of the upper abdomen and thorax, magnetic resonance imaging of the brain, whole‐body bone scintigraphy, fiberoptic bronchoscopy, and tissue histology.

All patients underwent either lobectomy or pneumonectomy as the primary surgical intervention with systematic lymph node dissection or selective lymph node dissection. Some patients required reconstruction of part of the left atrium or a large blood vessel at the time of the initial surgery.

The TCGA database contained clinical and genetic data from 494 patients with LUSCC, including 403 patients with stage T1‐T2 and 91 patients with stage T3‐T4 disease. The stage T1‐T2 patients included 270 cases without and 133 cases with lymph node metastasis. The stage T3‐T4 patients included 51 cases without and 40 cases with lymph node metastasis.

### 2.2 Study variables

Patient information, including survival time, demographic information (age and gender), the location of lung cancer, examination of regional nodes, lymph node metastasis, survival information, and living conditions, were obtained from the inpatient and The Cancer Genome Atlas (TCGA) databases. These characteristics were classified by categorical variables (eg, age, gender, the location of lung cancer, smoking history, lymph node metastasis, and degree of differentiation) using univariate and multivariate analysis. Age was classified into two groups (ie, ≤65 and >65 years old). Smoking history was classified into never‐smokers and smokers.

### 2.3 Statistical analysis

Statistical analysis was performed using SPSS version 20.0. The Kaplan‐Meier method was used to assess overall survival (OS) of patients with and without lymph node metastasis (LM and NLM, respectively). The two groups were compared using the log‐rank test. The Pearson's Chi‐square test was used to analyze the relationships between lymph node involvement and clinicopathologic variables. A two‐sided *P*‐value of .05 was considered statistically significant.

### 2.4 TCGA database analysis

We obtained clinical characteristics and genetic data for LUSCC patients from the TCGA database and excluded patients with undefined T or N staging. The data were then divided into two groups, T1‐T2 with lymph node metastasis group and T3‐T4 without lymph node metastasis and subjected to enrichment analysis for differentially expressed genes.

### 2.5 Preprocessing of RNA‐Seq data

TCGA‐Assembler software was used to analyze the LUSCC RNA‐Seq data from the TCGA database. Data for a total of 494 LUSCC patients were analyzed, including 270 NLM and 133 LN patients in the stage T1‐T2 group, and 51 NLM and 40 LN patients in the stage T3‐T4 group.

### 2.6 DEG screening

The edgeR package (Bioconductor) was used to identify differentially expressed genes (DEGs) between the LUSCC LM and LUSCC NLM, in T1‐2 group and T3‐4 group, respectively.^13^ The multi‐test package was used to determine the false discovery rate (FDR) and adjusted *P*‐values. The criteria for DEG screening were FDR < 0.05 and |log2‐fold change (FC)| > 1; FC = gene expression value for LUSCC with LM/gene expression value for LUSCC with NLM.

### 2.7 Construction of co‐expression network

The EBcoexpress package (Bioconductor) was used to obtain correlations between DEGs. A co‐expression network was constructed using DEG‐DEG pairs with correlation coefficients (|r|)> 0.6.

### 2.8 Functional and pathway enrichment analyses of DEGs

Gene annotation information was downloaded from the Gene Ontology (GO) Consortium (http://geneontology.org/). GO identifiers were further divided into cellular components (CC), biological processes (BP), and molecular function (MF) categories. We used the GO identifier to annotate the DEGs. Functional and Kyoto Encyclopedia of Genes and Genomes (KEGG) pathway enrichment of the DEGs in co‐expression networks were performed using DAVID^14^ and KOBAS 2.0^15^ based on a hypergeometric algorithm. The threshold for these analyses was set at *P* < .05. The result was visualized using FUNRICH (http://www.funrich.org/).

### 2.9 Protein‐protein interaction network (PPI network)

To further analyze the protein‐protein interaction network constructed by differential genes. We entered the differential genes into the multiple proteins list on the http://string-db.org/cgi/input.pl. We set organism to *Homo sapiens*, hid disconnected nodes in the network, and set the minimum required interaction score to highest confidence (0.900).

### 2.10 Small‐molecule drug analysis of DEGs

Based on the Connectivity Map database (https://portals.broadinstitute.org/cmap/),^16^ small‐molecule drug analysis was performed to determine the functional relationship between bioactive small‐molecule drugs and the DEGs. A correlation coefficient (|score|) > 0.3 was used as the evaluation criterion of this experiment.

## 3 RESULTS

### 3.1 Patient characteristics

Our retrospective study included a total of 170 patients. The clinical and pathological characteristics are presented in Table 1. The median patient age was 62 years (range, 39‐77 years). Most patients were male (85.3%). At the time of diagnosis, the majority of patients (40.6%) had an Eastern Cooperative Oncology Group Performance Status (ECOG‐PS) score of 3; no patients had an ECOG‐PS score of 4.

**Table 1:** Clinical features of SCC patients

Features	No. of Patients	%
Age (y)		
Median	62	
Range	39‐77	
Sex		
Male	145	85.3
Female	25	14.7
Smoking index (pack‐years)		
>400	99	58.2
≤400	71	41.8
Tumor location		
RUL	64	37.6
RML	11	6.5
RLL	26	15.3
LUL	48	28.2
LLL	21	12.4
Complications		
Yes	112	65.9
No	58	34.1
ECOG‐PS		
0	30	17.6
1	18	10.6
2	53	31.2
3	69	40.6
Tumor size (cm)		
<3	33	19.4
≥3	137	80.6
T stage		
1	15	8.8
2	77	45.3
3	26	15.3
4	52	30.6
N stage		
0	81	47.6
1	35	20.6
2	50	29.4
3	4	2.4
Surgery		
Lobectomy	159	93.5
Pneumonectomy	11	6.5
Lymph node dissection		
Systematic	158	92.9
Lymph node sampling	12	7.1
Residual tumor		
Yes	35	20.6
No	135	79.4
Vascular invasion		
Yes	35	20.6
No	135	79.4
Pleural effusion		
Yes	26	15.3
No	144	84.7

Abbreviations: LLL, left lower lobe; LUL, left upper lobe; RLL, right lower lobe; RML, right middle lobe; RUL, right upper lobe.

A total of 159 patients (93.5%) underwent lobectomy, and 11 underwent pneumonectomy (6.5%). Systematic lymph node dissection was performed in 158 patients (92.9%), while 12 patients underwent mediastinal lymph node sampling (7.1%).

All 170 patients had primary LUSCC, including 92 stage T1‐T2 patients and 78 stage T3‐T4 patients. There were 35 patients in the stage T1‐T2 group and 51 in the stage T3‐T4 group who had lymph node metastasis. Presence of lymph node metastasis were positively correlated with TNM staging regardless of whether they were stage T1‐T2 or T3‐T4 (*P*‐value = .002 and .013, respectively). Age was also correlated with T1‐T2 stage lymph metastasis (*P* = .002) (Table 2). These results were consistent with the TCGA database analysis (*P* < .001 [TNM stage] and .002 [age]) (Table 3).

**Table 2:** Clinicopathological characteristics of LUSC patients with or without LM

	T1‐T2 stage patients (n = 92)			T3‐T4 stage patients (n = 78)		
Characteristics	Without metastasis n = 57	With metastasis n = 35	Pearson's Chi‐square test (*P*‐value)	Without metastasis n = 27	With metastasis n = 51	Pearson's Chi‐square test (*P*‐value)
Age (%)	Median (Range) 62 (46‐77)			Median (Range) 62 (39‐75)		
≤65	30 (52.6)	27 (77.1)	.019	18 (66.7)	40 (78.4)	.285
>65	27 (47.4)	8 (22.9)		9 (33.3)	11 (21.6)	
Gender (%)						
Male	44 (77.2)	32 (91.4)	.096	24 (88.9)	45 (88.2)	>.999
Female	13 (22.8)	3 (8.6)		3 (11.1)	6 (11.8)	
TNM stage (%)						
I + II	57 (100)	15 (42.9)	<.001	10 (37.0)	0 (0)	<.001
III + IV	0 (0)	20 (57.1)		17 (63.0)	51 (100)	
Tumor location (%)						
LUL	14 (24.6)	6 (17.1)	.226	11 (40.7)	17 (33.3)	.911
LLL	9 (15.8)	5 (14.3)		2 (7.4)	5 (9.8)	
RUL	23 (40.4)	13 (37.1)		9 (33.3)	19 (37.3)	
RML	0 (0)	3 (8.6)		2 (7.4)	6 (11.8)	
RLL	11 (19.3)	8 (22.9)		3 (11.1)	4 (7.8)	
Smoker history (%)						
Never‐smokers	23 (40.4)	19 (54.3)	.25	10 (37)	19 (37.3)	>.999
Smokers	34 (59.6)	16 (45.7)		17 (63)	32 (62.7)	

Abbreviations: LLL, left lower lobe; LUL, left upper lobe; RLL, right lower lobe; RML, right middle lobe; RUL, right upper lobe

**Table 3:** Clinicopathological characteristics of LUSC patients with or without lymph metastasis from TCGA

	T1‐T2 stage patients (n = 403)			T3‐ T4 stage patients (n = 91)		
Characteristics	Without metastasis n = 270	With metastasis n = 133	Pearson's Chi‐square test (*P*‐value)	Without metastasis n = 51	With metastasis n = 40	Pearson's Chi‐square test (*P*‐value)
Age (%)	Median (Range) 68 (39‐90)			Median (Range) 70 (45‐84)		
≤65	89 (33.0)	62 (46.6)	.025	20 (39.2)	17 (42.5)	.481
>65	176 (65.2)	68 (51.1)		31(60.8)	22 (55.0)	
Unknown	5 (1.9)	3 (2.2)		0 (6.0)	1 (2.5)	
Gender (%)						
Male	199 (73.7)	98 (73.7)	>.999	38 (74.5)	30 (75)	>.999
Female	71 (26.3)	35 (26.3)		13 (25.5)	10 (25)	
TNM stage (%)						
I + II	270 (100)	99 (74.4)	<.001	39 (76.5)	0 (0)	<.001
III + IV	0 (0)	34 (25.6)		12 (23.5)	40 (100)	
Smoker history (%)						
Never‐smokers	8 (3.0)	4 (3.0)	.855	3 (5.9)	2 (5)	.965
Smokers	258 (95.6)	126 (94.7)		45 (88.2)	36 (90)	
Unknown	4 (1.5)	3 (2.3)		3 (5.9)	2 (5)	
Ethnicity (%)						
Asian	4 (1.5)	2 (1.5)	.186	1 (2)	1 (2.5)	.687
White	196 (72.6)	87 (65.4)		37 (72.5)	26 (65)	
Black or African American	18 (6.7)	6 (4.5)		2 (3.9)	4 (10)	
Unknown	52 (19.3)	38 (28.6)		11 (21.6)	9 (22.5)	

Abbreviations: LN, lymph node; LUSC, lung squamous cell carcinoma; TCGA, The Cancer Genome Atlas.

### 3.2 Lymph node metastasis can seriously affect lung cancer patient prognosis

**Figure 1:**
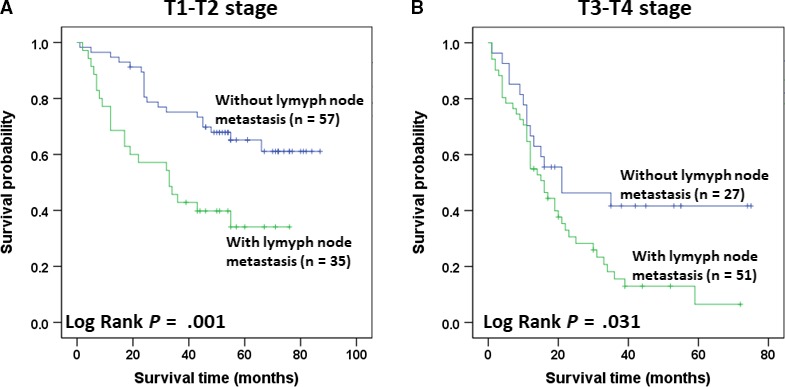
Kaplan‐Meier survival curves for different stages of LUSCC with and without lymph node metastasis. A, Kaplan‐Meier survival curves for overall survival (OS) between stage T1‐T2 patients without or with LM (n = 57 and 35, respectively). The log‐rank value (Mantel‐Cox) was 10.155, *P* = .001. B, Kaplan‐Meier survival curves for OS between 27 T3‐T4 patients without LM and 51 T3‐T4 patients with LM. The log‐rank value (Mantel‐Cox) was 4.674, *P* = .031

The T1‐T2 stage cases were divided into groups with and without lymph node metastasis. The T3‐T4 stage patients were similarly divided. We used Kaplan‐Meier survival curves to analyze the OS between these sets of groups as shown in Figure 1. There were significant differences between the lymph node metastasis and no lymph node metastasis groups in both the mean and median survival times (*P* = .031). The mean and median survival times of the lymph node metastasis group were significantly shorter than those of the no lymph node group. The results for the univariate and multivariate Cox regression survival analyses for the OS in SCC are shown in Table 4. In the univariate analysis, lymph node metastasis and tumor location predicted a worse OS. However, there were no significant differences in the OS based on gender, age, or smoking history. In the multivariate analysis, the data were adjusted for gender, age, smoking history, lymph node metastasis, and tumor location. The results showed that lymph node metastasis was an independent predictor of OS (Table 5).

**Table 4:** Tumor lymph metastasis associated with overall survival in NSCLC

Pathological type	Factors	n	MST (mo)	MST (mo)	*P* †
T1‐T2 stage n = 92	LM	35	39.71	33.00	‐‡
	NLM	57	65.62	—	
T3‐T4 stage n = 78	LM	51	21.85	16.00	.031
	NLM	27	39.04	21.00	

Abbreviations: LM, lymph node metastasis; MST, mean survival time; NLM, no lymph node metastasis.

^†^ *P*: Univariate analysis log‐rank *P* value.

^‡^ ‐: Most patients were still alive, and the MST could not be calculated.

**Table 5:** Univariate and multivariate Cox hazard regression

Pathological type	Characteristics	Univariate Hazard Ratio (95% CI)	*P* value	Multivariate Hazard Ratio (95% CI)	*P* value
T1‐T2 stage n = 92	Gender	0.391‐1.985	.760	0.399‐2.349	.943
	Age	0.955‐1.034	.752	0.561‐2.331	.711
	Smoking history	0.445‐1.505	.518	0.510‐1.922	.977
	Lymph node metastasis	1.403‐4.760	.002	1.402‐5.417	.003
	Tumor location	1.058‐1.667	.015	1.070‐1.732	.012
T3‐T4 stage n = 78	Gender	0.553‐2.710	.618	0.463‐2.794	.780
	Age	0.975‐1.040	.672	0.979‐1.043	.519
	Smoking history	0.682‐2.005	.569	0.679‐2.009	.575
	Lymph node metastasis	1.040‐3.407	.037	1.034‐3.495	.039
	Tumor location	0.783‐1.149	.588	0.768‐1.168	.609

### 3.3 DEGs between LUSCC with and without lymph node metastasis

We used RNA‐Seq data from the TCGA database (total of 19 754 genes) to further clarify the DEGs in stage T1‐T2 and T3‐T4 patients with lymph node metastasis compared to those without lymph node metastasis. For the stage T1‐T2 patients, a total of 353 significant DEGs (FDR < 0.05 and |log2 FC| > 1) were identified between the LM and NLM groups (176 upregulated, 177 downregulated). For the stage T3‐T4 patients, we found 127 significant DEGs (FDR < 0.05 and |log2 FC| > 1) between the LM and NLM groups (93 upregulated, 34 downregulated). A list of the top ten upregulated and downregulated genes for the stage T1‐T2 and T3‐T4 patients are presented in Complementary Tables 1 and 2, respectively.

**Complementary Table 1:** The top ten different upregulated and downregulated genes between T1‐T2 stage metastatic and non‐metastatic patients in TCGA‐SCC

Genes name	logFC	logCPM	*P* Value	FDR	Regulated
*KRT33A*	1.91932	1.170314	5.78E‐12	1.13E‐07	Upregulated
*TAC1*	4.993138	1.83953	1.35E‐28	2.67E‐24	Upregulated
*HHATL*	3.10014	−1.07393	1.21E‐22	2.39E‐18	Upregulated
*CPS1*	1.634023	4.783704	7.17E‐10	1.40E‐05	Upregulated
*MYOC*	1.473326	−1.08875	9.29E‐07	0.018026	Upregulated
*CASR*	1.715779	−1.32471	3.73E‐12	7.33E‐08	Upregulated
*GUCA2B*	2.594166	−3.56981	1.41E‐12	2.78E‐08	Upregulated
*MS4A12*	2.459106	−3.67545	1.58E‐09	3.10E‐05	Upregulated
*MPP4*	1.134123	−0.3291	4.12E‐08	0.000803	Upregulated
*HBQ1*	1.486433	−2.03354	1.86E‐09	3.65E‐05	Upregulated
*ZMYND10*	−1.1228	2.015232	6.58E‐08	0.001283	Downregulated
*CALCR*	−1.48878	−0.79119	4.75E‐11	9.32E‐07	Downregulated
*PON1*	−1.28726	−0.8732	7.64E‐07	0.014834	Downregulated
*ASB4*	−1.6684	0.178399	1.13E‐10	2.21E‐06	Downregulated
*SLC7A14*	−2.13177	0.172122	3.64E‐07	0.007086	Downregulated
*STMN4*	−1.79836	−1.31754	1.74E‐08	0.000339	Downregulated
*VSIG2*	−1.10892	2.772969	3.00E‐08	0.000586	Downregulated
*C8B*	−1.45914	−0.88102	1.60E‐06	0.030901	Downregulated
*GUCA1A*	−1.85823	0.702034	1.76E‐13	3.46E‐09	Downregulated
*POU1F1*	−1.55124	−3.39582	1.50E‐08	0.000292	Downregulated

Abbreviations: logCPM, log2‐counts‐per‐million; logFC, log2‐fold change; TCGA‐SCC, The Cancer Genome Atlas Lung Squamous Cell Carcinoma.

**Complementary Table 2:** The top ten different upregulated and downregulated genes between T3‐T4 stage patients with metastatic and non‐metastatic patients in TCGA‐SCC

Genes name	logFC	logCPM	*P*‐value	FDR	Regulated
*CALCR*	2.077648	−0.89763	4.79E‐08	0.000943	Upregulated
*FMO3*	2.752062	5.794534	2.21E‐09	4.36E‐05	Upregulated
*MYOC*	5.795709	0.537182	3.03E‐16	5.98E‐12	Upregulated
*NNAT*	2.939417	2.519234	4.63E‐12	9.15E‐08	Upregulated
*CTNNA2*	3.755315	0.103113	3.90E‐07	0.00766	Upregulated
*FGF10*	3.250026	−0.42509	5.12E‐10	1.01E‐05	Upregulated
*SEZ6L*	3.842698	3.01891	2.63E‐07	0.00518	Upregulated
*CPNE6*	2.378734	−2.41143	1.39E‐06	0.027217	Upregulated
*NTSR1*	5.307006	2.302344	2.62E‐16	5.18E‐12	Upregulated
*PROKR2*	4.86744	−2.25973	5.37E‐07	0.01056	Upregulated
*SLC22A16*	−2.93798	0.399663	4.24E‐08	0.000836	Downregulated
*CASR*	−5.10319	0.442459	6.19E‐12	1.22E‐07	Downregulated
*FSTL3*	−1.55333	5.673753	7.33E‐07	0.01441	Downregulated
*LUZP4*	−4.00178	−2.73786	1.88E‐06	0.036902	Downregulated
*TRPA1*	−3.38389	4.679141	2.21E‐08	0.000436	Downregulated
*PPP1R17*	−6.97007	0.20054	9.29E‐11	1.83E‐06	Downregulated
*CHRNA2*	−3.4004	−2.21239	1.91E‐07	0.003754	Downregulated
*KRT36*	−3.31532	1.936803	1.77E‐06	0.034822	Downregulated
*FTHL17*	−6.61953	0.142442	3.54E‐08	0.000697	Downregulated
*FXYD2*	−3.46056	1.926779	8.58E‐09	0.000169	Downregulated

Abbreviations: FDR, false discovery rate; logCPM, log2‐counts‐per‐million; logFC, log2‐fold change; TCGA‐SCC, The Cancer Genome Atlas Lung Squamous Cell Carcinoma.

**Figure 2:**
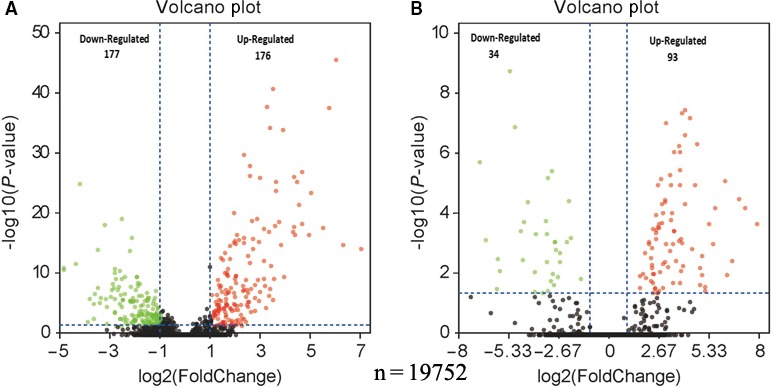
Volcano plot comparing DEGs of patients with or without LM. A, Volcano plot comparing the DEGs of stage T1‐T2 patients with or without LM in the TCGA‐SCC. B, Volcano plot comparing the DEGs of stage T3‐T4 patients with or without LM in the TCGA‐SCC

**Figure 3:**
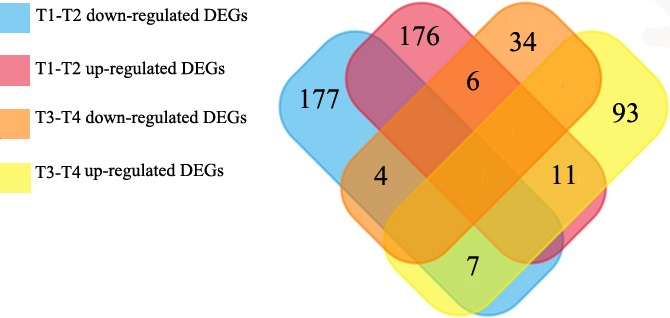
Comparison of DEGs. Using the analysis software, we found that 176 genes were upregulated and 177 genes downregulated in patients with T1‐T2 stage disease with LM compared to patients without LM. In addition, 93 upregulated and 34 downregulated DEGs were found in T3‐T4 stage patients from the TCGA database. Twenty‐eight of these genes were in the intersection

The differential genes were mapped into a volcano plot (Figure 2). Among the DEGs, there were 28 genes in the intersection (11 upregulated, four downregulated). Thirteen genes showed different trends between the lymph node metastasis and no lymph node metastasis groups (Figure 3).

### 3.4 Functional annotation of DEGs

**Figure 4:**
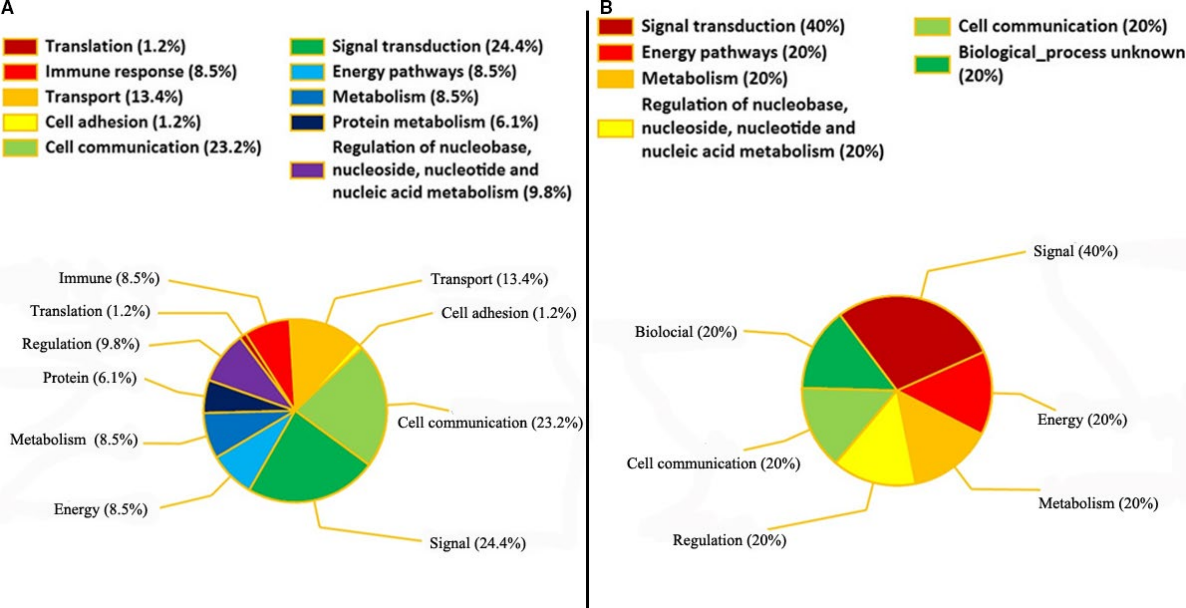
GO functions involving DEGs in the co‐expression network. A, GO functions for T1‐T2 co‐expression DEGs. B, GO functions for T3‐T4 co‐expression DEGs. The percentage represents the percentage of DEGs in a specific term compared to all DEGs. GO, Gene Ontology; DEGs, differentially expressed genes

After GO functional annotation, 353 T1‐T2 stage DEGs were predicted to participate in five categories each of CC, MF, and BP (Figure 4A,C,E). The CC categories were mainly associated with the extracellular, plasma membrane, cytoplasm, exosomes, and nucleus. The MF categories mainly consisted of transporter activity, G‐protein coupled receptor activity, transcription factor activity, extracellular matrix structural constituents, and catalytic activity. The BP categories were mainly associated with signal transduction, cell communication, transport, cell growth or maintenance, and cell adhesion. The cell adhesion categories only included upregulated DEGs. For the T3‐T4 stage DEGs, 127 were also predicted to participate in five categories each of CC, MF, and BP (Figure 4B,D,F). The CC categories were mainly associated with the extracellular, plasma membrane, cytoplasm, nucleus, and integral to plasma membrane. The MF categories included transporter activity, transcription factor activity, structural molecule activity, structural constituent of cytoskeleton, and growth factor activity. The BP categories consisted of signal transduction, cell communication, transport, cell growth or maintenance, and energy pathways. The categories for growth factor activity and transporter activity only included upregulated DEGs.

### 3.5 Enrichment analysis of DEGs in co‐expression network

**Figure 5:**
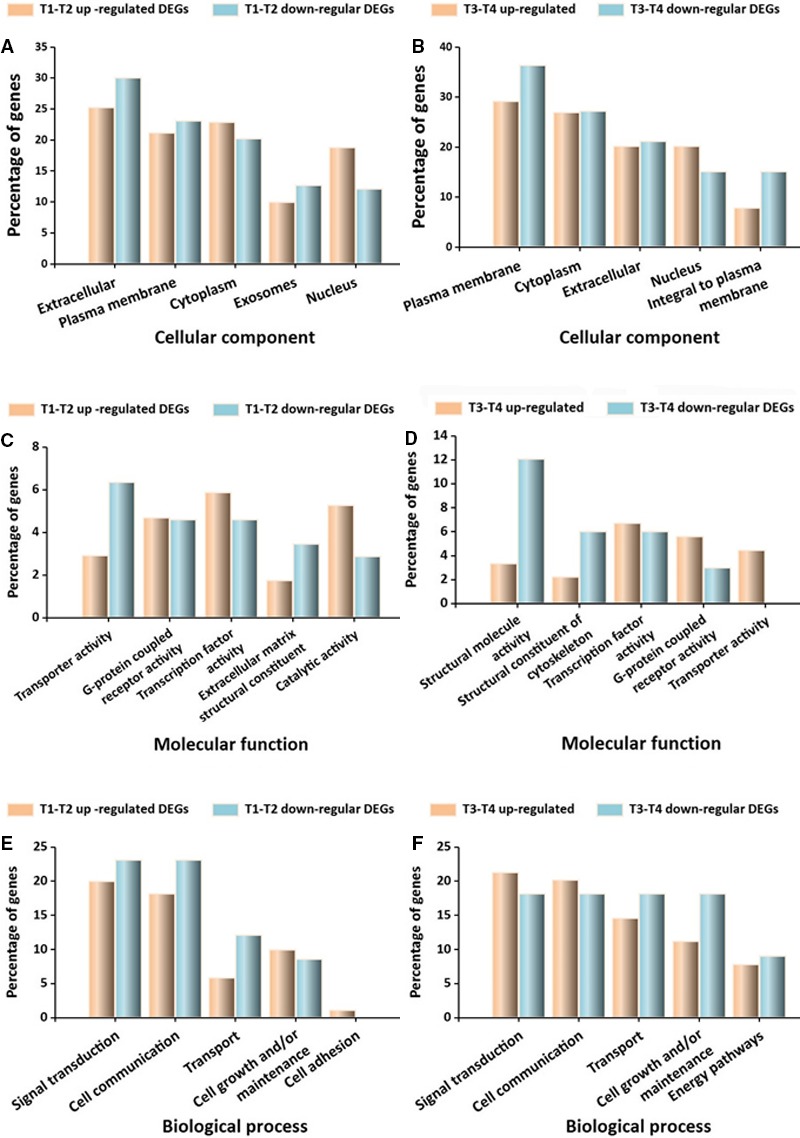
GO annotations for the DEGs. A and B, DEGs in cellular component categories. C and D, DEGs in molecular function categories. E and F, DEGs in biological process categories. DEGs: differentially expressed genes. Left vertical axis: percentage of DEGs involved in a specific term out of all DEGs

After DEGs GO functional analyses, we further analyzed the DEGs perspective in the co‐expression network and did the functional enrichment. A total of 84 genes were found in T1‐T2 DEGs, which constituted a gene co‐expression network. T1‐T2 stage DEGs in the co‐expression network were significantly enriched for 12 functions, including translation, immune response, transport, cell adhesion, cell communication, signal transduction, energy pathways, metabolism, protein metabolism, biological process unknown, regulation of nucleobase, nucleoside, nucleotide and nucleic acid metabolism, cell growth, and maintenance (*P* < .05). A total of five DEGs genes in T3‐T4 DEGs were involved in co‐expression network, which were significantly enriched in six functions, including signal transduction, energy pathways, metabolism, regulation of nucleobase, nucleotide and nucleic acid metabolism, cell communication, and unknown biological processes (*P* < .05) (Figure 5).

### 3.6 KEGG enrichment analysis

To further study the signaling pathways for the DEGs, we performed KEGG enrichment analysis using the KEGG database. The results identified a total of 21 signal pathways associated with the T1‐T2 DEGs with maturity‐onset diabetes of the young ranked first (adjusted *P* = 1.63E‐06) (Complementary Table 3). There were seven signaling pathways associated with the T3‐T4 DEGs and the neuroactive ligand‐receptor interaction ranked first (Complementary Table 4).

**Complementary Table 3:** KEGG enrichment analysis of T1‐T2 DEGs

ID	Description	*P*‐value	Adjust *P*‐value	Included genes
hsa04950	Maturity onset diabetes of the young	1.09E‐08	1.63E‐06	*NEUROG3/ HNF4A/ SLC2A2/ NEUROD1/ PAX4/ HNF1A/ RFX6*
hsa04951	Neuroactive ligand‐receptor interaction	1.58E‐06	0.000119	*CHRNA2/ CHRM2/ CHRNA6/ FSHB/ CGA/ HTR5A/ CALCR/ GRM3/ OPRD1/ CSH1/ RXFP1/ PRLHR/ GHSR*
hsa04952	Metabolism of xenobiotics by cytochrome P450	5.75E‐06	0.000287	*CYP1A2/ CYP2B6/ CYP1A1/ UGT2B11/ CYP2A6/ UGT2B10/ GSTM5*
hsa04953	Taste transduction	1.26E‐05	0.000471	*TRPM5/ TAS2R42/ PKD1L3/ CALHM1/ HTR3D/ SCN3A/ HTR3E*
hsa04954	Steroid hormone biosynthesis	1.82E‐05	0.000546	*CYP1A2/ CYP1A1/ UGT2B10/ UGT2B11/ HSD3B1/ AKR1C4*
hsa04955	Retinol metabolism	3.31E‐05	0.000826	*CYP1A2/ CYP2B6/ CYP1A1/ UGT2B10/ CYP2A6/ UGT2B11*
hsa04956	Drug metabolism ‐ cytochrome P450	4.52E‐05	0.000968	*CYP1A2/ CYP2B6/ UGT2B10/ CYP2A6/ UGT2B11/ GSTM5*
hsa04957	Proximal tubule bicarbonate reclamation	7.81E‐05	0.001465	*CA4/ FXYD2/ PCK1/ SLC38A3*
hsa04958	Complement and coagulation cascades	9.14E‐05	0.001523	*VTN/ MASP1/ C8B/ CPB2/ C4BPB/ SERPINA5*
hsa04959	Chemical carcinogenesis	0.000111	0.001663	*CYP1A2/ CYP1A1/ UGT2B10/ CYP2A6/ UGT2B11/ GSTM5*
hsa04960	Protein digestion and absorption	0.000179	0.002443	*COL2A1/ CPB1/ CPB2/ MEP1A/ FXYD2/ COL9A1*
hsa04961	Tryptophan metabolism	0.000539	0.006739	*DDC/ CYP1A2/ TPH1/ CYP1A1*
hsa04962	Fat digestion and absorption	0.000588	0.006743	*APOA1/ PLA2G2A/ MTTP/ APOB*
hsa04963	Nitrogen metabolism	0.000629	0.006743	*CA4/ CA5A/ CPS1*
hsa04964	ECM‐receptor interaction	0.000914	0.009124	*RELN/ VTN/ COL9A1/ COL2A1/ TNR*
hsa04965	PI3K‐Akt signaling pathway	0.000973	0.009124	*EIF4E1B/ CHRM2/ COL2A1/ KIT/ VTN/ TNR/ RELN/ CSH1/ COL9A1/ PCK1*
hsa04966	Ovarian steroidogenesis	0.001175	0.010365	*CGA/ HSD3B1/ CYP1A1/ FSHB*
hsa04967	Caffeine metabolism	0.00148	0.012337	*CYP1A2/ CYP2A6*
hsa04968	Serotonergic synapse	0.00337	0.026602	*HTR3D/DDC/TPH1/HTR5A/HTR3E*
hsa04969	PPAR signaling pathway	0.004117	0.030876	*APOA1/APOA2/APOA5/PCK1*
hsa04970	Pyruvate metabolism	0.005896	0.042113	*ACOT12/LDHAL6A/PCK1*

**Complementary Table 4:** KEGG enrichment analysis of T3‐T4 DEGs

ID	Description	*P*‐value	Adjusted *P*‐value	Included genes
hsa04080	Neuroactive ligand‐receptor interaction	2.74E‐07	2.38E‐05	*NEUROG3/ HNF4A/ SLC2A2/ NEUROD1/ PAX4/ HNF1A/ RFX6*
hsa04742	Taste transduction	0.000152	0.006632	*CHRNA2/ CHRM2/ CHRNA6/ FSHB/ CGA/ HTR5A/ CALCR/ GRM3/ OPRD1/ CSH1/ RXFP1/ PRLHR/ GHSR*
hsa04020	Calcium signaling pathway	0.000272	0.007887	*CYP1A2/ CYP2B6/ CYP1A1/ UGT2B11/ CYP2A6/ UGT2B10/ GSTM5*
hsa00982	Drug metabolism ‐ cytochrome P450	0.001428	0.031059	*TRPM5/ TAS2R42/ PKD1L3/ CALHM1/ HTR3D/ SCN3A/ HTR3E*
hsa04610	Complement and coagulation cascades	0.002074	0.036086	*CYP1A2/ CYP1A1/ UGT2B10/ UGT2B11/ HSD3B1/ AKR1C4*
hsa04974	Protein digestion and absorption	0.002966	0.043003	*CYP1A2/ CYP2B6/ CYP1A1/ UGT2B10/ CYP2A6/ UGT2B11*
hsa04972	Pancreatic secretion	0.003537	0.043965	*CYP1A2/ CYP2B6/ UGT2B10/ CYP2A6/ UGT2B11/ GSTM5*

### 3.7 PPI analysis

**Figure 6:**
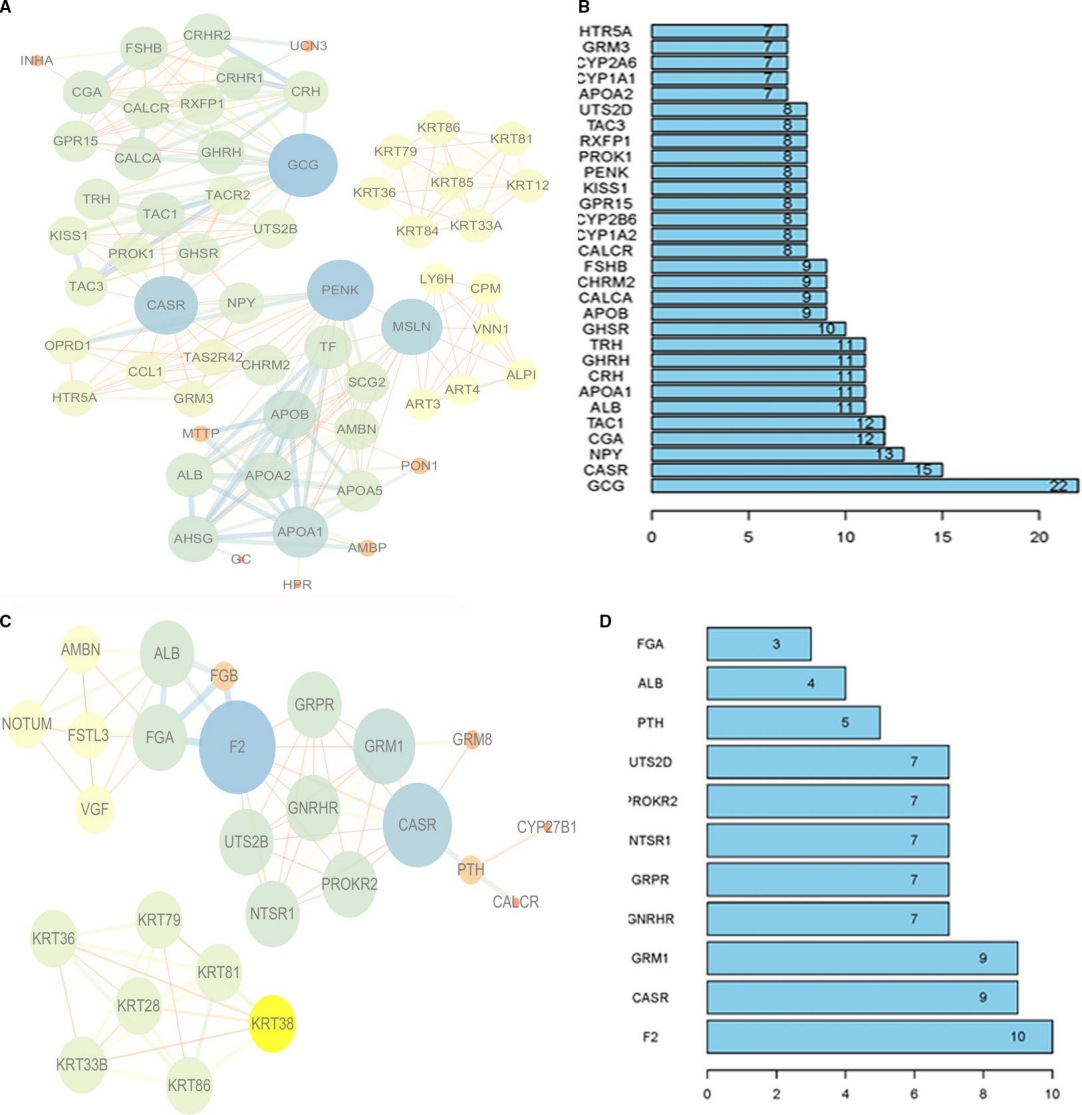
Protein‐protein interaction network of DEGs. A, PPI network of T1‐T2 DEGs. B, The number of genes associated with other T1‐T2 DEGs. C, PPI network of T3‐T4 DEGs. D, The number of genes associated with other T1‐T2 DEGs. Circles represent genes, lines represent interactions between genes, and the results inside circles represent the structure of the proteins. The color of the thread represents different types of evidence for an interaction between the proteins (red, fusion evidence; green, neighborhood evidence; blue, co‐occurrence evidence; purple, experimental evidence; yellow, text mining evidence; light blue, database evidence; black, co‐expression evidence)

We conducted further interactive network analysis of the DEGs using STRING version 5.5. The established protein‐protein interaction networks for the T1‐T2 and T3‐4 DEGs are presented in Figure 6. We found that there were a total of 20 genes located at the core of the protein‐protein interaction network for the T1‐T2 DEGs, including *GCG, CASR, NPY, CGA, TAC1, ALB, APOA1, CRH, CHRH, TRH, GHSR, APOB, CALCA, CHRM2, FSHB, CALCR, CYP1A2, CYP2B6, GPR15, KISS 1, PENK, PROK1, RXFP1, TAC3, UTS2D, APOA2, CYP1A1, CYP2A6, GRM3,* and *HT25A* (number of links to other genes ≥7) as shown in Figure 6A,B. There were 11 genes in the core of the network for the T3‐T4 stage DEGs, which consisted of *F2, CASR, GRM1, GNRHR, GRPR, NTSR1, PROKR2, UTS2B, PTH, ALB,* and *FGA* (number of links to other genes ≥3) as presented in Figure 6C‐D.

### 3.8 Small‐molecule drug analysis of DEGs

The Connectivity Map (Cmap) is a collection of genome‐wide transcriptional expression data from cultured human cells treated with bioactive small molecules and simple pattern‐matching algorithms, which together enable the discovery of functional connections between drugs, genes, and diseases through the transitory feature of common gene‐expression changes. We used Cmap to identify small‐molecule drugs that had a positive or negative correlation with the DEGs from in this study. The range of the correlation scores was 1 to −1. A score <0 indicated that a drug could inhibit tumor lymph node metastasis whereas a score >0 revealed that a drug could promote tumor lymph node metastasis. We found that for T1‐T2 stage LUSCC, only one small‐molecule drug (cefotiam) could counteract the genetic changes that were associated with lymph node metastases (Complementary Table 5). For T3‐4 stage LUSCC, 13 drugs were negatively or positively correlated with lymph node metastases. Among them, seven drugs might inhibit lymph node metastases (raloxifene, iproniazid, exisulind, arachidonyltrifluoromethane, 16‐phenyltetranorprostaglandin E2, econazole, and fluoxetine). In contrast, five drugs might promote lymph node metastases (AH‐6809, ticarcillin, 5255229, mesoridazine, and stachydrine) (Complementary Table 5). These data suggest that these drugs should be used with caution in LUSCC patients.

**Complementary Table 5:** Small‐molecule drugs correlated with T1‐T2 stage or T3‐T4 stage lymph node metastases DEGs

Stage	Small molecule drugs	Mean correlation score	*P*‐value
T1‐T2 DEGs	cefotiam	−0.365	0.00024
T3‐T4 DEGs	raloxifene	−0.367	0.00226
	AH‐6809	0.781	0.00258
	iproniazid	−0.349	0.00965
	exisulind	−0.678	0.01235
	arachidonyltrifluoromethane	−0.617	0.01286
	16‐phenyltetranorprostaglandin E2	−0.661	0.01373
	ticarcillin	0.478	0.0152
	econazole	−0.574	0.01866
	5255229	0.375	0.02821
	mesoridazine	0.361	0.02912
	stachydrine	0.331	0.03815
	fluoxetine	−0.336	0.04645

The mean score changes from 1 to −1. A score <0 means the change in the direction of the gene expression values caused by the drug are opposite to the change in the direction of the gene expression values in lymph node metastasis. Low scores represent high anti‐LM. In contrast, a score >0 means the change in the direction of the gene expression values caused by the drug are the same as the change in the direction of the gene expression values in lymph node metastasis (eg, the drug might promote LM. In addition, high scores represent high lymph node metastasis‐promoting effects. DEGs: differentially expressed genes.

## 4 DISCUSSION

Locally advanced lung cancers invading the heart, great vessels, trachea, or vertebrae have historically been classified as unresectable. These tumors were usually treated with palliative chemotherapy or radiation, alone or in combination. However, advances in surgical techniques that go far beyond standard surgery have been challenging this dogma for the last three decades.^17^ Currently, patients with local invasion but without lymphatic and distant metastasis, who undergo surgical resection within a multimodality treatment regimen, have the best chance for cure. However, does early T stage mean early N stage? There are still many early T stage patients with lymphatic metastasis, who not only need radical resection and systemic lymphadenectomy, but also chemotherapy, radiation, and targeted therapy. Therefore, it is essential to understand the risk factors associated with lymphatic spread. What are the genetic differences in patients with and without early‐stage T lymph node metastasis? Why does a large proportion of patients present with lymph node metastasis at an early stage? What are the differences and similarities in gene expression between early and advanced lymph node metastases?

In the present study, age was significantly correlated with stage T1‐T2 lymph metastasis, which was consistent with the TCGA analysis. In the early disease stages, younger patients (<65) were more likely to have lymphatic metastasis. These findings were similar to those for melanoma, in which older melanoma patients had lower rates of sentinel lymph node metastases.^18^ In thyroid papillary microcarcinomas, large‐volume LM was more frequently found in young (<40 years) and male patients.^19^ These findings support the notion that surgery and systemic lymphadenectomy rather than selective lymph node dissection may be favored in young clinically LN‐negative LUSCC patients as a primary therapeutic option.

Due to the decreasing cost of current sequencing technology, the ability to explore the genetic characteristics of tumors is becoming widely available. An important question is how to use this abundance of genetic information judiciously with a focus on genes that have been previously associated with a specific cancer type. In this study, we retrospectively examined data from both our cohort of patients and the TCGA database, and found that in addition to age, the expression of specific genes in primary tumor tissue correlated with lymph node metastasis in early T stage (T1 and T2) LUSCC and local advanced T stage (T3 and T4) LUSC without lymphatic metastasis. A total of 353 significant DEGs were identified between the lymph node metastasis and no lymph node metastasis groups for the T1‐T2 stage patients, including 176 upregulated and 177 downregulated DEGs. In addition, there were 93 upregulated and 177 downregulated DEGs identified for the T3‐T4 group. There were 28 genes at the intersection. We believe that these 28 genes may be the driver genes for SCC lymph node metastasis. In particular, genes with similar tendencies in both groups may promote LUSC lymph node metastasis, and genes with different tendencies in both groups may be associated with local invasion without metastasis. Among these genes, the human albumin gene (ALB) and the extracellular calcium‐sensing receptor (CASR) genes are located at the core of the protein‐protein interaction network. ALB (GenBank mRNA RefSeq: NM_000477.6) is considered relevant to congenital analbuminaemia, a very rare autosomal recessive disorder with an estimated prevalence of less than 1 in 1 million).^20^ There have been no reports that have associated ALB with cancer lymph node metastasis. CaSR belongs to class C of the GPCR, which signals in response to Ca^2+^ and other ligands, such as gadolinium, polypeptides, and certain antibiotics.^21^ CaSR can promote the development of bone metastasis in both renal cell carcinoma^22^ and breast cancer.^23^ Based on the KEGG Enrichment analysis, a total of 21 signaling pathways are involved in the T1‐T2 DEGs, of which the maturity onset diabetes of the young may be of most interest. Its ID is hsa04950, and its related genes include *NEUROG3, HNF4A, SLC2A2, NEUROD1, PAX4, HNF1A, and RFX6*. The discovery of this pathway is consistent with recent studies that have demonstrated that altered glucose metabolism is closely related to the occurrence and development of lung cancer,^24^, ^25^, ^26^ and may suggest that it might also be related to lymph node metastasis. Furthermore, combined treatment with metformin and gefitinib overcomes the primary resistance to EGFR‐TKIs via targeting the IGF‐1R signaling pathway,^27^ which is also related to glucose metabolism. However, this conclusion does not apply to advanced lung cancer. There were also seven signaling pathways involved with the T3‐T4 DEGs with the neuroactive ligand‐receptor interaction (ID: hsa04080) ranked first. They are potentially targeted by drugs, such as hydroxyzine.^28^

The epithelial‐mesenchymal transition (EMT) is an important process for tumor invasion and metastasis mediated by complex regulatory mechanisms. EMT is a key event in the transformation of tumor cells from an epithelial‐like to interstitial phenotype, which can promote tumor invasion, metastasis, and drug resistance. The hallmark of EMT is the alteration of epithelioid cell markers in tumor cells, such as down‐regulation of E‐cadherin expression and concomitant upregulation of mesenchymal cell markers, such as N‐cadherin, vimentin, and snail transcription factor family members.^29^, ^30^ Attenuation of E‐cadherin reduces intercellular adhesion and increases cell motility, allowing cells to invade surrounding tissues.^31^, ^32^ Multiple signaling pathways are involved in the EMT process in tumor cells, such as Wnt, transforming growth factor‐β (TGF‐β), and Notch signaling pathways.^33^ However, we do not know the relationship among metastasis and DGEs, EMT respectively. Therefore, exploring the molecular mechanism of EMT and identifying genes that regulate this process will have important implications.^34^ The study is the first time we try to explore the ideal gene and its up‐ or down‐regulation can further affect the early or late metastasis of tumors. So we did not have a specific selection of EMT signaling pathways. Actually, we plan to identify all DEGs may cause premature tumor metastasis first and explore their function in the EMT signaling pathway, tumor microenvironment and epigenetic changes in further research. Further analysis will focus on whether specific genes are directly related to the EMT‐related pathways, and whether other pathways could directly affect invasion and metastasis by LUSCC.

In summary, our TCGA database analysis showed that there were some significant DEGs (eg *ALB* and *CaSR)* associated with early T stage lymphatic metastasis and local invasion without lymphatic metastasis. Furthermore, signaling pathways, such as hsa04080 (neuroactive ligand‐receptor interaction) and hsa04950 (maturity onset diabetes of the young), mediate the effects of these DEGs. In future research, we will collect long‐term follow‐up and additional genetic data to validate the functions of these genes. Further analysis of the DEGs identified 84 co‐expressed genes among the T1‐T2 and T3‐T4 DEGs that likely play a significant role in gene regulation. Small‐molecule drug analysis, initially suggested that some drugs probably target the DEGs in different stage squamous cell lung cancer, but unfortunately, these drugs are not significantly associated with these small‐molecules (0.8 > |score| > 0.3). Thus, there are few targeted drugs currently available to squamous cell lung cancer patients.

## 5 CONCLUSION

Lymph node metastasis plays a key role in the treatment response and prognosis of LUSCC patients. In the early T stages, younger patients (<65) have a stronger tendency for lymphatic metastasis. Surgery and systemic lymphadenectomy rather than selective lymph node dissection may be favored for young clinically LN‐negative LUSCC patients as a primary therapeutic option. This unprecedented systems biology analysis of squamous cell lung cancer with or without lymph node metastasis showed statistically significant enrichment of LUSCC genes and identified genetic features of lymphatic metastasis and local invasion by LUSCC. Long‐term follow‐up and additional genetic data are needed to validate gene function in LUSCC. The identified genes may have the potential to predict prognosis and serve as therapeutic targets.

## CONFLICT OF INTEREST

The authors declare that they have no conflict of interest.

## AUTHORS' CONTRIBUTIONS

Ming Dong, Hao Gong, and Tong Li helped to collect the data and analyze the GEO data. Xin Li, Jinghao Liu, Hongbing Zhang, Minghui Liu and Gang Chen helped to collect the data from our department and discussed the manuscript. Ming Dong, Hongyu Liu, and Jun Chen analyzed the data and wrote the manuscript. All authors read and approved the final manuscript.

## ETHICS APPROVAL AND CONSENT TO PARTICIPATE

This study was approved by the Ethical Review Committee of Tianjin Medical University General Hospital. All biological samples were obtained with patients informed written consent. The study conforms to the Declaration of Helsinki.

## CONSENT FOR PUBLICATION

Not applicable.

## Data Availability

Data sharing is not applicable to this article as no datasets were generated or analyzed during the current study.
